# Children With Cystic Fibrosis Are Infected With Multiple Subpopulations of *Mycobacterium abscessus* With Different Antimicrobial Resistance Profiles

**DOI:** 10.1093/cid/ciz069

**Published:** 2019-01-26

**Authors:** Liam P Shaw, Ronan M Doyle, Ema Kavaliunaite, Helen Spencer, Francois Balloux, Garth Dixon, Kathryn A Harris

**Affiliations:** 1 UCL Genetics Institute, University College London, London; 2 Nuffield Department of Medicine, John Radcliffe Hospital, Oxford; 3 Department of Microbiology, Virology and Infection Control; 4 National Institute for Health Research Biomedical Research Centre; 5 Paediatric Respiratory Medicine and Lung Transplantation, Great Ormond Street Hospital National Health Services Foundation Trust, London, United Kingdom

**Keywords:** lung transplant, whole-genome sequencing, within-patient diversity, macrolides, physiological niches

## Abstract

**Background:**

Children with cystic fibrosis (CF) can develop life-threatening infections of *Mycobacterium abscessus*. These present a significant clinical challenge, particularly when the strains involved are resistant to antibiotics. Recent evidence of within-patient subclones of *M. abscessus* in adults with CF suggests the possibility that within-patient diversity may be relevant for the treatment of pediatric CF patients.

**Methods:**

We performed whole-genome sequencing (WGS) on 32 isolates of *M. abscessus* that were taken from multiple body sites of 2 patients with CF who were undergoing treatment at Great Ormond Street Hospital, United Kingdom, in 2015.

**Results:**

We found evidence of extensive diversity within patients over time. A clustering analysis of single nucleotide variants revealed that each patient harbored multiple subpopulations, which were differentially abundant between sputum, lung samples, chest wounds, and pleural fluid. The sputum isolates did not reflect the overall within-patient diversity and did not allow for the detection of subclones with mutations previously associated with macrolide resistance (*rrl* 2058/2059). Some variants were present at intermediate frequencies before the lung transplants. The time of the transplants coincided with extensive variation, suggesting that this event is particularly disruptive for the microbial community, but the transplants did not clear the *M. abscessus* infections and both patients died as a result of these infections.

**Conclusions:**

Isolates of *M. abscessus* from sputum do not always reflect the entire diversity present within the patient, which can include subclones with differing antimicrobial resistance profiles. An awareness of this phenotypic variability, with the sampling of multiple body sites in conjunction with WGS, may be necessary to ensure the best treatment for this vulnerable patient group.

## Teaser

Children with cystic fibrosis undergoing lung transplants harbor multiple subpopulations of *Mycobacterium abscessus.* Subpopulations can have different antimicrobial resistance genotypes. Sputum isolates do not reflect the genetic diversity within a patient.

**(See the Editorial Commentary by Griffith on pages 1687–9.)**

*Mycobacterium abscessus* is a nontuberculous mycobacteria that has recently emerged as a major pathogen in cystic fibrosis (CF) patients [1]. Infection with *M. abscessus* is associated with poor clinical outcomes, particularly in conjunction with lung transplantation [2]. Treatment is challenging, due to the intrinsic resistance of *M. abscessus* to many classes of antibiotics [3], along with certain genotypes that drastically alter the efficacy of antibiotics [4]. The antimicrobial resistance (AMR) profile of isolates is highly relevant for treatment, but current diagnostic work mainly uses isolates from sputum, which may not reflect the full range of genetic diversity within the patient and, therefore, may fail to recover the true AMR profile.

Minority variants from whole-genome sequencing (WGS) have been used to infer the presence of multiple subpopulations (subclones) in longitudinal sputum isolates of *M. abscessus* [5]. However, whether patients harbor further unsampled genetic diversity remains an open question. The lung is known to be capable of harboring considerable pathogen diversity in chronic infections. For example, *Mycobacterium tuberculosis* infections exist as multiple subpopulations with different AMR profiles [6, 7]. The potential relevance of this genetic diversity for treatment is not yet known.

**Figure 1.**
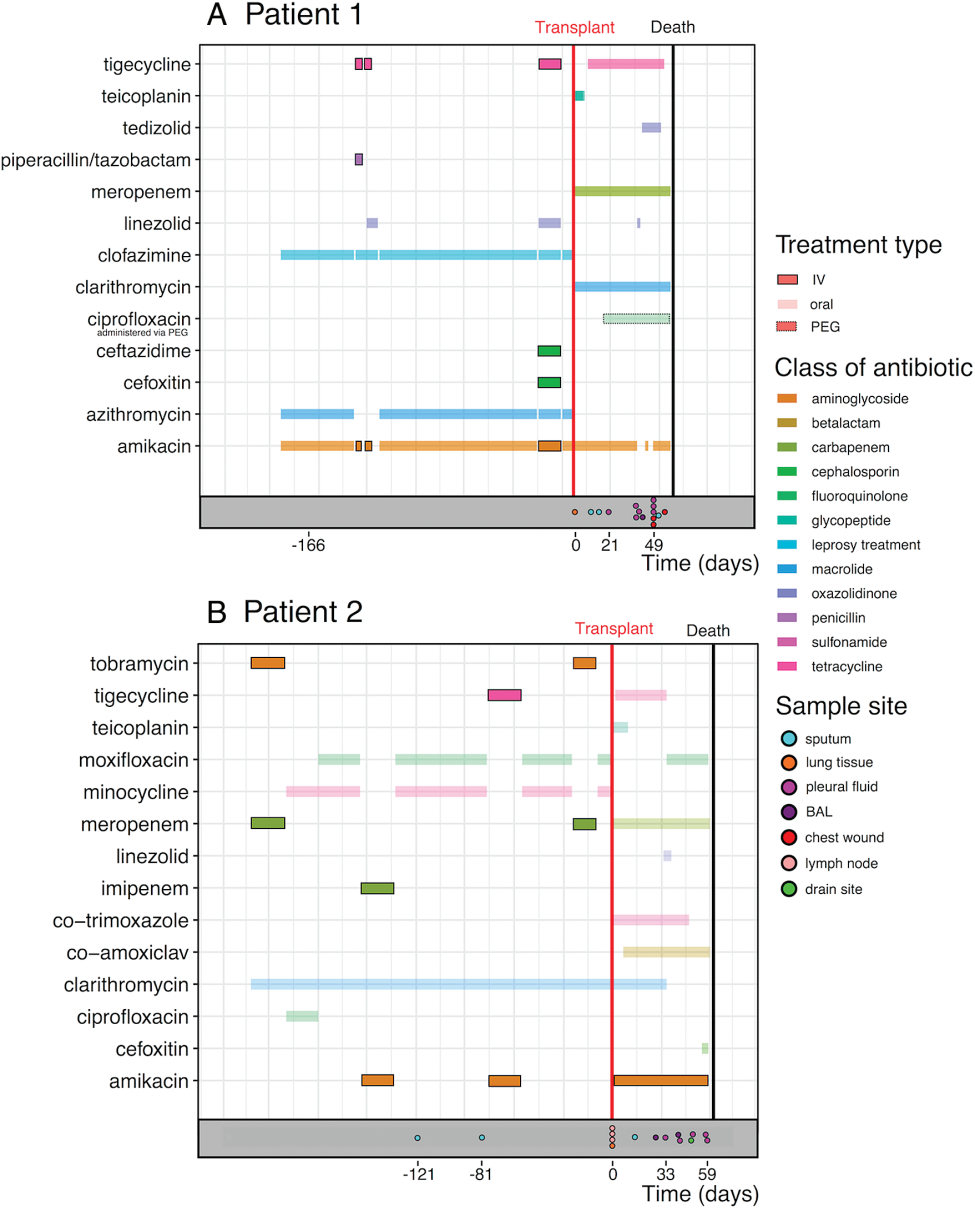
Overview of the sampling scheme and antibiotic treatment. The treatment regimes included intravenous antibiotics (boxed colored lines) and oral antibiotics (faint colored lines) for both patients during the 6-month period prior to their lung transplant (red vertical line). The sampling scheme is represented at the bottom of each panel (colored points). Abbreviations: BAL, bronchoalveolar lavage; IV, intravenous; PEG, percutaneous endoscopic gastrostomy.

For this reason, we investigated longitudinal isolates from 2 patients infected with *M. abscessus subsp. abscessus *who were undergoing lung transplants at Great Ormond Street Hospital (Figure 1) and identified variable genomic positions within samples (single nucleotide variants [SNVs]). By including isolates not only from sputum samples, but also biologically important compartments such as pleural fluid, lung tissue, and swabs from chest wounds, we aimed to establish the extent and significance of the within-patient variation in *M. abscessus* for this vulnerable group.

## METHODS

### Patient Cohort and Sample Collection

Great Ormond Street Hospital is a large, regional center for pediatric CF patients and is the largest pediatric lung transplant center in the United Kingdom. The 2 patients in this study (Patient 1 and Patient 2) were from other CF centers and were seen at Great Ormond Street Hospital for a transplant assessment, during the lung transplant procedure, and posttransplant (Table 1). Both patients in this study underwent regular respiratory microbiological diagnostic investigations, including specific stains and cultures for mycobacteria on sputum pretransplant and posttransplant and for explanted lung tissue, bronchoalveolar lavage, pleural fluid, and clamshell incision wound swabs posttransplant. All further microbiological investigations were carried out on “sweeps” from pure culture plates. All M. abscessus isolates cultured in our laboratory are identified to the subspecies level by polymerase chain reaction (PCR) amplification and sequencing of the hsp65 and rpoB genes, and by inducible macrolide resistance predicted by PCR amplification and sequencing of the erm(41) gene, as previously described [8]. Variable nucleotide tandem repeat (VNTR) profiles were obtained for selected isolates, as previously described [9]. Phenotypic sensitivity data was obtained from the mycobacterial reference laboratory (Table 3).

**Table 1.** Summary Statistics for the *De novo* Reference Genomes of the 2 Patients

	Patient 1	Patient 2
Total bases	5 172 759	5 275 491
Mean depth of coverage	25.3X	74.1X
Number of contigs	28	46

References were assembled for the first temporal sample from each patient (patient_1_S1 and patient_2_S1; see Methods).

Demographic and clinical data were extracted from the Patient Administration System and microbiological data from the Laboratory Information Management system (OMNI-client ISS) using structured query language databases and Excel spreadsheets. Additional sources of information included CF and transplantation databases. Details of the antimicrobial therapies administered prior to transplantation were provided by the referring CF centers. All investigations were performed in accordance with the Hospitals Research governance policies and procedures.

### Whole-genome Sequencing

DNA extraction was performed on 16 isolates from Patient 1 and 16 isolates from Patient 2 (Figure 1; Table 2), as previously described [9], with the addition of a bead beating step. The total DNA concentration was determined using the Qubit high-sensitivity assay kit (Thermofisher) and a sequencing library was prepared from 50 ng of DNA using the Nextera Library Preparation kit (Illumina). The post-PCR clean-up was carried out using Ampure XP beads (Beckman). The library size was validated using the Agilent 2200 TapeStation with the Agilent D1000 ScreenTape System, and 150 bp paired-end reads were sequenced on the Illumina NextSeq 550 system. The raw sequencing reads have been deposited on the European Nucleotide Archive (Study Accession PRJEB28875), as have 2 assemblies used as de novo references (see below).

**Table 2.** Patient Clinical Data

Patient	*M. abscessus* Subspecies	Sex	CF Genotype	Date of Last Smear-positive Sample	Date of Last *M. abscessus* Isolate
Patient 1	abscessus	Female	F508del/F508del	−166 days	+56 days
Patient 2	abscessus	Male	F508del/W1282X	−81 days	+59 days

Dates are relative to the patient’s day of transplant.

Abbreviations: CF, cystic fibrosis; *M. abscessus*, *Mycobacterium abscessus*.

### Sequence Data Analysis

Initial VNTR typing, carried out as described previously [9, 10], suggested the possibility of mixed infections, based on results intermediate between VNTR I and the closely related VNTR I* profile (differing at 1 locus). A preliminary mapping of all isolates to the standard *M. abscessus* strain ATCC 19977 chromosome (NCBI Accession: CU458896.1) showed that the mean coverage at 10X was ~91%, contrasted with >99% for a representative set of VNTR II isolates from another patient sequenced with the same protocol (not shown). In order to ensure we captured as much genetic diversity as possible, we therefore adopted a hybrid de novo and mapping approach. We selected the first isolate (temporally) for each patient and performed de novo assembly with SPAdes v3.10.0 with the --careful switch and, otherwise, default parameters [11]. After removing contigs with <10 000 bases to exclude small, mobile, genetic elements, this first de novo assembly was used as a new reference to map raw reads from other isolates using bwa mem v0.7.12 with default parameters [12]. This produced de novo references for Patient 1 and Patient 2, containing 5.17 Mb and 5.28 Mb, respectively (Table 1). Contigs were reordered against the *M. abscessus* ATCC 19977 chromosome using Mauve v2.4.0 (2015-02-25) [13].

### Variant Identification and Clustering Analysis

In brief, the mapping file was sorted and indexed using picard v1.130; then, Genome Analysis Toolkit v3.30 was used to create a combined variant call format file for each patient. Each position required a mapping depth >30 in all samples from a patient to be included in the downstream analysis. We manually inspected the “self-mapping” of the reads from the first temporal sample to its own de novo assembly using Integrative Genomics Viewer v2.4.10 [14] to identify small regions where the mapping was problematic. We removed SNVs within isolated regions where the self-mapping had unexpected peaks in coverage (Patient 1: contigs 12 [12 575-13 519 bp] and 19 [96 916–97 032 bp]; Patient 2: contig 17 [12 718-12 770 bp]), as well as SNVs where the reference allele fraction from the self-mapping reads was <5%.

As noted by Bryant et al [5], patterns of linkage of variants in *M. abscessus* can be suggestive of the existence of subpopulations. We aimed to establish a conservative lower bound for the number of clonal subpopulations within a patient, inferring their existence from the linkage patterns of variant frequencies across all samples. Patterns of linkage disequilibrium can also occur, due to recombination; therefore, we attempted to remove local recombination in our analysis. Using the SNVs obtained via the mapping and filtering methods described above, we hierarchically clustered SNVs using Ward’s minimum variance criterion [15], applied to Euclidean distances between allele frequencies, with a dissimilarity threshold of 1 to define clusters. We removed clusters containing <4 SNVs. We also removed putative local recombination regions by removing clusters where the SNVs were distributed within a total range <100 000 bp (~2% of the *M. abscessus* genome).

In general, the inference of haplotype frequencies using variant frequencies from short sequencing reads for a microbial population undergoing recombination is a complex problem [16]. However, as we are not attempting to comment on abundances of subpopulations, but only on their presence, we did not need to infer haplotype frequencies. Observing *n* distinct clusters of variants within a patient over time (ie, allele frequencies that covary in step with each other) means that there must be at least *n* bacterial haplotypes producing these patterns within the population. This fact holds even when recombination is present. Therefore, observing distinct clusters of linked variants tells us that distinct subpopulations of *M. abscessus* exist within individual samples.

## RESULTS

### Individuals Harbor Extensive Variation

**Figure 2.**
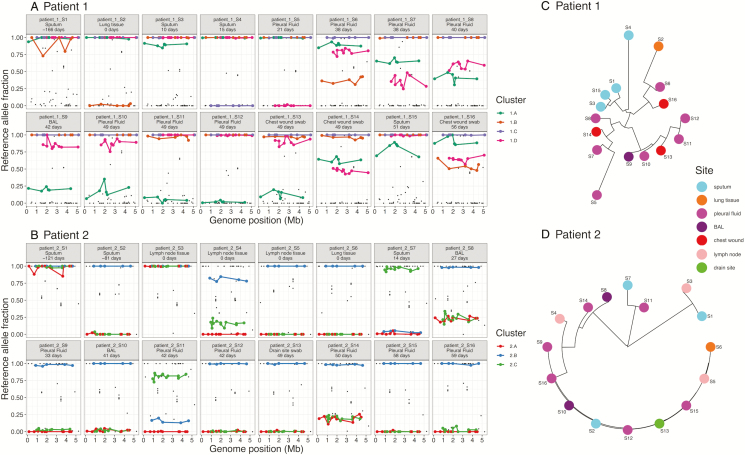
Linkage patterns of SNVs across samples suggest the presence of closely related subpopulations within patients. *A* and *B*, SNVs were grouped into clusters (colors) using an unsupervised clustering technique, showing clear patterns of abundance across samples (see Methods). The genome position was inferred by ordering *de novo* contigs against the *Mycobacterium abscessus* ATCC 19977 reference genome. *C* and *D*, The midpoint-rooted neighbor-joining trees are based on Euclidean distances between samples, using these clustered SNVs to show this variation within patients over time (numbers) and body sites (colors). Abbreviations: BAL, bronchoalveolar lavage; SNV, single-nucleotide variants.

We observed multiple positions in the *M. abscessus* genome, which varied between different isolates within a patient over time (total variable positions used for clustering across all isolates: Patient 1, 54 positions; Patient 2, 64 positions), although the isolates remained highly similar, on average, and were clearly the same infecting strain (mean inter-isolate SNV distances: Patient 1, 2.07 +/- 0.92 SNVs; Patient 2, 1.96 +/- 1.81 SNVs). Subsets of these SNVs showed patterns of linkage across the *M. abscessus* genome (Figure 2A). Similar, clustered patterns of linked SNVs could also arise due to recombination, but clustered SNVs were widely spread across the genome, suggesting the presence of multiple subclones. Even if recombination were present, leading to mixtures of clusters (ie, different haplotypes), then the observed abundance patterns still required multiple subclones. A neighbor-joining tree produced from the distances between isolates, based on these allele frequencies, also suggested multiple subclones (Figure 2B).

### Within-patient Variation Includes Antimicrobial Resistance Mutations

**Figure 3.**
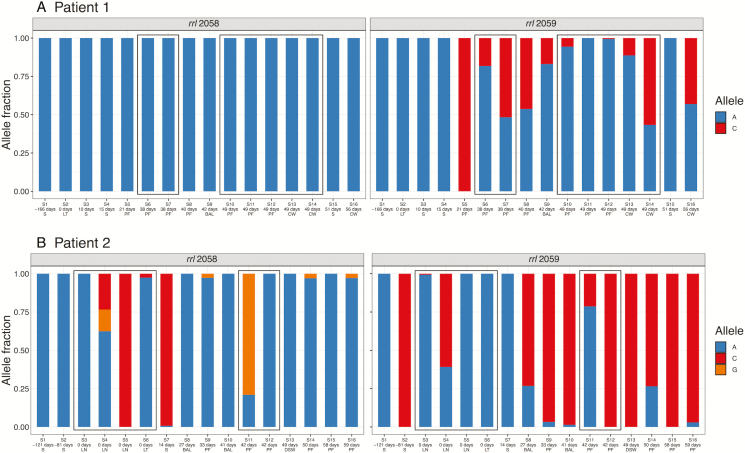
Variants in the *rrl* gene (23S rRNA) arose during treatment and were present in isolates from concurrent samples. Relative allele fractions at these positions show that, although the initial sputum isolate was susceptible for both patients, resistance appeared to develop during treatment. Samples are ordered by time, with boxes indicating samples taken on the same day. Samples taken on the day of transplant are shown in bold text. Here, following the usual convention for the *rrl* gene, we use *Escherichiacoli* numbering. Positions 2058 and 2059 in *E. coli* correspond to 2269 and 2270 in *Mycobacterium abscessus.* Abbreviations: BAL, bronchoalveolar lavage; CW, chest wound; DSW, drain site swab; LN, lymph node; LT, lung tissue; PF, pleural fluid; S, sputum.

Macrolide resistance in *M. abscessus* is driven by mutations at established positions in the *rrl* gene. Both patients received macrolides almost continuously throughout the 6 months prior to their transplants (Figure 1). While initial isolates taken earlier in treatment were susceptible, we observed that resistance alleles at these positions (C/G) increased in abundance over time (Figure 3). Notably, all sputum isolates from Patient 1 showed a susceptible allele at position 2059, whereas isolates from the pleural fluid and clamshell incision wound swabs carried a resistance allele (A2059C; Figure 3A). There was also substantial variation within sets of isolates taken on the same day. For example, 3 isolates from different lymph node samples taken on the day of transplant for Patient 2 showed completely different macrolide resistance profiles, most strikingly at position 2058 (Figure 3B). These positions were not among the SNVs clustered into subpopulation structure clusters in Patient 2, but *rrl* 2059 was part of cluster 1.D in Patient 1, demonstrating that these resistance alleles can arise spontaneously, but also persist as linked to the genetic background.

**Figure 4.**
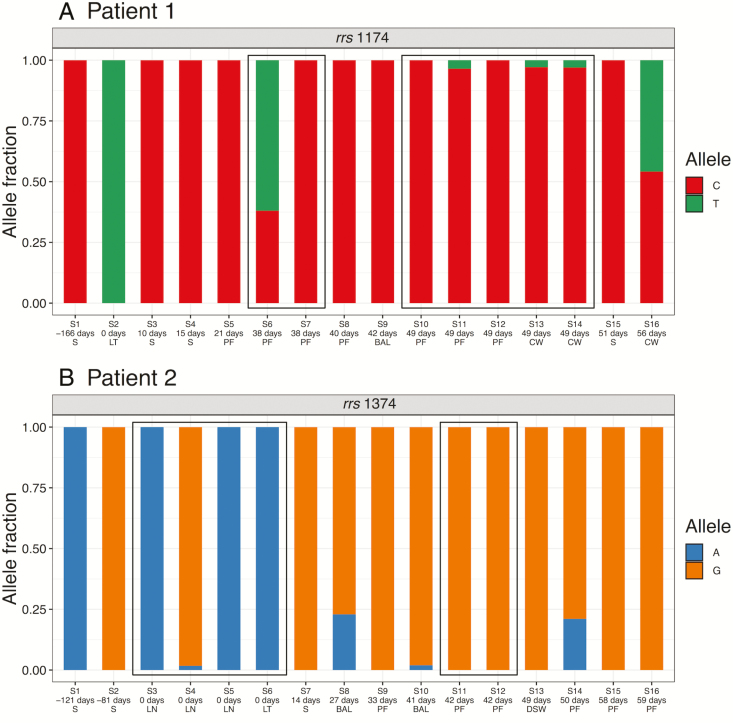
Variants in the *rrs* (16S rRNA) gene over the course of treatment. Patient 1: position 1174. Patient 2: position 1374, previously associated with amikacin resistance (see text). The numbering is relative to ATCC 19977 reference. Abbreviations: BAL, bronchoalveolar lavage; CW, chest wound; DSW, drain site swab; LN, lymph node; LT, lung tissue; PF, pleural fluid; S, sputum.

We also observed variable positions in the *rrs* gene in both patients (Figure 4). The *rrs* gene codes for 16S ribosomal RNA (rRNA) and is often a site of the emergence of aminoglycoside resistance (eg, to amikacin), particularly in the last few 100 bp of the gene, where the secondary structure of the rRNA can be affected by multiple mutations [17]. The de novo reference assembly for the gene in both patients was identical to the previously characterized sequence from amikacin-resistant *M. abscessus* [17, 18], which was consistent with the measured AMR phenotype of the first sputum sample in Patient 2, but not Patient 1 (Table 3). Subsequently, an isolate from the lung tissue of Patient 1, taken on the day of transplant, had a different allele at position 1174 (C→T; Figure 4) and was partially resistant when phenotyped (Table 3), suggesting this mutation may have been involved in this resistance. In Patient 2, we observed both A and G at position 1374, corresponding to the A1400G mutation, which confers high-level amikacin resistance in *M. tuberculosis* [19]. The G allele was dominant by the end of treatment, suggesting that it may have conferred higher resistance and/or been an important compensatory mutation.

**Table 3.** Routine Microbiology Data About Isolates

Sample	Body Site	Date Isolated	Colony Morphotype	VNTR Profile	Phenotypic Susceptibility
patient_1_S1	Sputum	-166 days	Rough	I	Amikacin-S; Cipro-R; Clarithromycin-R; Doxycycline-R; Augmentin-R
patient_1_S2	Lung tissue	0 days	Rough	I	Amikacin-PR; Cipro-R; Clarithromycin-R; Doxycycline-R; Linezolid-R; Co-trem-R; Cefotaxime-R
patient_1_S3	Sputum	10 days	Smooth	I	n/a
patient_1_S4	Sputum	15 days	Rough	I	n/a
patient_1_S5	Pleural fluid	21 days	Smooth	I	n/a
patient_1_S6	Pleural fluid	38 days	Rough	I*	n/a
patient_1_S7	Pleural fluid	38 days	Rough	I*	n/a
patient_1_S8	Pleural fluid	40 days	Smooth	I*	n/a
patient_1_S9	BAL	42 days	Smooth	I*	n/a
patient_1_S10	Pleural fluid	49 days	Smooth	I*	n/a
patient_1_S11	Pleural fluid	49 days	Smooth	I*	n/a
patient_1_S12	Pleural fluid	49 days	Smooth	I*	n/a
patient_1_S13	Chest wound swab	49 days	Smooth	I / I*	n/a
patient_1_S14	Chest wound swab	49 days	Smooth	I	n/a
patient_1_S15	Sputum	51 days	Smooth	I / I *	n/a
patient_1_S16	Chest wound swab	56 days	Rough	I	n/a
patient_2_S1	Sputum	−121 days	Rough	n/a	Amikacin-R; Cipro-R; Clarithromycin-R; Doxycycline-R; Linezolid-R; Co-trem-R; Cefotaxime-R
patient_2_S2	Sputum	−81 days	Smooth	I	n/a
patient_2_S3	Lymph node tissue	0 days	Rough	I	n/a
patient_2_S4	Lymph node tissue	0 days	Smooth	n/a	n/a
patient_2_S5	Lymph node tissue	0 days	Rough	I	n/a
patient_2_S6	Lung tissue	0 days	n/a	I	n/a
patient_2_S7	Sputum	14 days	Smooth	n/a	n/a
patient_2_S8	BAL	27 days	Smooth	I	n/a
patient_2_S9	Pleural fluid	33 days	Smooth	I	n/a
patient_2_S10	BAL	41 days	Smooth	I / I*	n/a
patient_2_S11	Pleural fluid	42 days	Smooth	I	n/a
patient_2_S12	Pleural fluid	42 days	Smooth	I	Amikacin-R; Ciprofloxacin-R; Clarithromycin-R; Doxycycline-R; Linezolid-R; Co-trimoxazole-R; Cefoxitin-R; Tobramycin-R; Moxyfloxacin-R
patient_2_S13	Drain site swab	49 days	Smooth	n/a	n/a
patient_2_S14	Pleural fluid	50 days	Smooth	n/a	n/a
patient_2_S15	Pleural fluid	58 days	Smooth	I	n/a
patient_2_S16	Pleural fluid	59 days	Smooth	n/a	n/a

Dates are relative to the date of transplant for each patient. An asterisk (*) indicates that the VNTR profile differed at 1 locus. Phenotypic susceptibility was available for a minority of isolates.

Abbreviations: BAL, bronchoalveolar lavage; n/a, data not available; -PR, partially resistant; -R, resistant; -S, susceptible; VNTR, variable nucleotide tandem repeat.

Other sites also showed high levels of variation in both patients. After sorting SNVs by the standard deviation of the reference allele fraction across isolates (Supplementary Dataset 1), the most highly variable position in Patient 1 was in the putative ferric uptake regulator FurB (MAB_1678c). This variant was present at high abundance in samples taken 49 days after transplant, with the reference allele fraction only present at <2% in 1 pleural fluid sample (Supplementary Dataset 1). Ferric uptake regulation has been associated with the virulence of pathogenic mycobacteria; in mycobacterial infections, the host response deprives the bacteria of iron to prevent replication [20]. Iron is important for growth and virulence in *M. abscessus* [21], with gallium used as a treatment because of its ability to inhibit iron-dependent enzymes [22]. The most variable position in Patient 2 was within a putative linoleoyl-coenzyme A (CoA) desaturase (MAB_2148), and the second most highly variable position in Patient 2 was within the cell division control protein 48 (MAB_0347). Population heterogeneity via asymmetric cell division has been suggested as a factor facilitating the survival of *M. tuberculosis* across host physiological niches [23], and the control of cell division is probably similarly important in the survival of *M. abscessus* across body sites. Although at different positions, both patients had a variable position within *erm(41)* (MAB_2297), which confers inducible resistance to macrolides [24].

### Sputum Samples Do Not Reflect Overall Within-patient Diversity

The first sample from each patient was from sputum. The frequencies of the reference alleles were significantly associated with body sites in Patient 2 (Kruskal-Wallis rank-sum test, *P *< .001), but not in Patient 1 (Kruskal-Wallis rank-sum test, *P *= .13). Reference allele frequencies were significantly higher in subsequent sputum isolates, compared to non-sputum isolates, for the majority of SNVs in both patients (Patient 1: 51/54 SNVs, with *P *< .05 after Benjamini-Hochberg correction; Patient 2: 61/64 SNVs, with *P *< .05 after Benjamini-Hochberg correction), suggesting that sputum isolates tended to be more similar to the initial sputum isolate used as a reference, even for Patient 1, where 3/3 subsequent sputum isolates were posttransplant (immunosuppressed). This also suggests that non-sputum isolates harbored additional diversity that was not well sampled using sputum.

## DISCUSSION

In this retrospective study, we sought to establish the extent of within-patient variability of *M. abscessus* in 2 patients who developed severe complications following lung transplants as part of treatment for CF. We used WGS to characterize this variability in isolates from longitudinal clinical samples, and were able to reveal patterns of linkage of SNVs that were consistent with the presence of multiple subpopulations within patients.

Isolates from the same patient were similar—for example, all within-patient inter-isolate distances were within the threshold of 25 SNVs that was previously suggested for inferring potential transmission events [25] and, while mixed populations could theoretically lead to different transmission inferences, the between-patient variation was significantly larger—but this does not mean that the variation is not clinically significant. We have demonstrated that within-patient variation can be biologically and clinically important. Notably, we observed that variation at *rrl* 2058/2059, associated with macrolide resistance, developed over the course of treatment. We observed extensive variation in isolates from Patient 2 prior to and on the day of transplant (ie, before the patient was immunosuppressed). Isolates from Patient 1 prior to and on the day of transplant displayed no variation at these positions, so presumably, the phenotypic macrolide resistance reported in these samples at this time was due entirely to the inducible resistance conferred by *erm(41)*. Nonetheless, *rrl* 2058/2059 variants were present in later isolates, suggesting that macrolide use still has a therapeutic impact, even in the presence of a functional *erm(41)* gene, and drives the selection of high-level macrolide resistance. We also observed variation in the *rrs* gene—another source of resistance to antibiotics which target ribosomal function, such as amikacin—as both previously recorded and novel positions, including an allele that rose in dominance over the course of treatment in Patient 2 (Figure 4).

When resistance to antibiotics is driven by point mutations at single positions, natural mutation rates will lead to the repeated presence of naturally occurring, resistant cells. Conservatively, taking values from the more slow-growing and non-recombining *M. tuberculosis,* a mutation rate of ~8 × 10^-9^ mutations per site per month [26] and a typical extracellular population of ~10^9^ cells [27] clearly means that, during treatment, a typical within-patient population will repeatedly give rise to cells with the *rrl* 2058 mutation (for example). Typically, such mutations have fitness costs, so remain at a low frequency, but in the presence of antibiotics, they rapidly achieve dominance. In *M. tuberculosis,* combination therapy is specifically designed to combat this selection of resistance. The strong resistance selection effect we observed for macrolides in *M. abscessus* highlights the weakness of current treatment regimens: particularly, the lack of good companion medications.

Based on this data, we would question how useful the phenotypic testing of isolates recovered from a limited number of sputum samples is for guiding antimicrobial therapy, as this strategy is unlikely to capture the diversity present in the full sample. Similarly, even though WGS is clearly a valuable tool, it may also fail to capture an accurate AMR profile if restricted to the analysis of sputum isolates. Previous studies on *M. tuberculosis* have shown that mycobacterial culture reduces the diversity recovered from sputum samples [28, 29]. It is, therefore, possible that the diversity found across different sample sites in this study may have been present in sputum samples, but was lost in the culture step. For *M. tuberculosis*, direct sequencing from sputum samples using capture-based enrichment methods has been shown to recover sample diversity not present in liquid culture [30]. By extension, it is possible that WGS, at a high depth, applied directly to sputum samples, could identify the variants detected between sample sites.

We observed highly variable positions in genes with direct relevance for the survival of *M. abscessus* across different physiological niches, such as the regulation of ferric uptake (MAB_1678c) and the control of cell division (MAB_0347). In a chronic infection, mycobacteria must cope with considerable host stresses, including iron starvation, which enables the persistence of *M. tuberculosis* in granulomas [31]. Phenotypic diversity due to expression can also occur: the colony morphotype (smooth or rough) has previously been linked to the phenotype, although both morphotypes appear to be capable of aggregation and intracellular survival [32]. Further diversity may come from the subpopulations harbored at different locations within the patient’s lungs. Regional selective pressures within the lungs have been shown to drive the diversification of *Pseudomonas aeruginosa,* another chronic CF pathogen, and we would expect similar dynamics for *M. abscessus.* In particular, different body regions may have different antibiotic antibiotic concentrations. It has been recently shown for *M. tuberculosis* that antibiotic concentrations vary across biopsy sites in cavities and that this variation is associated with different minimum inhibitory concentrations and resistance-associated variants [7]. It seems highly plausible that similar effects exist in *M. abscessus* infections, and this is an important area for further research.

Our findings suggest that the wider diversity present within patients chronically infected with *M. abscessus* is not well sampled with sputum, and that the body site influences the subpopulation structure. More widespread sampling of multiple body sites would provide a more accurate picture of the AMR profile of *M. abscessus* in a patient, and may be necessary to guide targeted antimicrobial therapy prior to a transplant. However, in practical terms, this would mean taking biopsies, which carries a significant clinical risk and would probably be unfeasible in patients awaiting transplants. An alternative solution to improve patient management before a transplant might rely on deep-sequencing of multiple sputum samples. Such a strategy might capture a sufficient fraction of the total within-patient diversity to provide accurate information about the presence of minor variants: in particular, those conferring AMR.

## Supplementary Data

Supplementary materials are available at *Clinical Infectious Diseases* online. Consisting of data provided by the authors to benefit the reader, the posted materials are not copyedited and are the sole responsibility of the authors, so questions or comments should be addressed to the corresponding author.

ciz069_suppl_Supplementary_Material

## Notes

***Financial support.*** This work was supported by the European Metrology Programme for Innovation and Research, cofinanced by the participanting states, and from the European Union’s Horizon 2020 research and innovation program. The study was also supported by the National Institute for Health Research Biomedical Research Centre at Great Ormond Street Hospital for Children National Health Service Foundation Trust; and University College London. L.P.S and F.B. acknowledge financial support from the Medical Research Council (grant MT/P007597/1).

***Potential conflicts of interest.*** All authors: No reported conflicts. All authors have submitted the ICMJE Form for Disclosure of Potential Conflicts of Interest. Conflicts that the editors consider relevant to the content of the manuscript have been disclosed.
